# Patients with Common Variable Immunodeficiency Complicated by Autoimmune Phenomena Have Lymphopenia and Reduced Treg, Th17, and NK Cells

**DOI:** 10.3390/jcm10153356

**Published:** 2021-07-29

**Authors:** Ewa Więsik-Szewczyk, Elżbieta Rutkowska, Iwona Kwiecień, Marcelina Korzeniowska, Dariusz Sołdacki, Karina Jahnz-Różyk

**Affiliations:** 1 Department of Internal Medicine, Pulmonology, Allergy and Clinical Immunology, Military Institute of Medicine, Szaserów 128, 04-141 Warsaw, Poland; mkorzeniowska1@wim.mil.pl (M.K.); dariusz.soldacki@gmail.com (D.S.); kjrozyk@wim.mil.pl (K.J.-R.); 2 Laboratory of Hematology and Flow Cytometry, Department of Internal Medicine and Hematology, Military Institute of Medicine, Szaserów 128, 04-141 Warsaw, Poland; erutkowska@wim.mil.pl (E.R.); ikwiecien@wim.mil.pl (I.K.); 3 Department of Clinical Immunology, Medical University of Warsaw, 02-691 Warsaw, Poland

**Keywords:** B cell maturation, CD4+ cells, T cell maturation, primary immune deficiency, autoimmunity, connective tissue diseases

## Abstract

Most patients with primary immune deficiency suffer from recurrent infections; however, paradoxical autoimmune phenomena can also manifest. The aim of this study was to identify immunological markers of autoimmune phenomena associated with common variable immunodeficiency (CVID). The study included 33 adults with CVID divided into two groups: (1) those with noninfectious autoimmune complications (CVID-C (*n* = 24)) and (2) those with only infectious symptoms (CVID-OI (*n* = 9)). Flow cytometry of peripheral blood was performed and compared with systemic lupus erythematosus (SLE) patients (*n* = 17) and healthy controls (*n* = 20). We found that all lymphocytes were lower in CVID-C and SLE. NK cells were lowest in CVID-C. Th17 cells were significantly reduced in CVID-C and SLE. Tregs were significantly lower in CVID-C and SLE. Bregs did not significantly differ between any groups. Class-switched memory B cells were significantly lower in CVID-C and CVID-OI. Lastly, plasmablasts were significantly higher in SLE. Among the T cell subsets, CVID-C patients had lower naive and recent thymic emigrant CD4+ T cells. In conclusion, reduced Treg, Th17, and NK cells are features of CVID with autoimmune complications, and class-switched memory B cells can help distinguish patients with different causes of autoimmunity. Future studies are needed to confirm whether reductions of Treg, Th17, and NK cells might be a biomarker of more complicated CVID cases.

## 1. Introduction

Common variable immunodeficiency (CVID) is the most common symptomatic primary antibody deficiency in adults. Most patients suffer from recurring respiratory tract infections; however, paradoxical autoimmunity, both systemic and organ related, is a secondary manifestation of CVID and affects 20–40% of cases [1]. Patients with a complicated CVID phenotype have the longest diagnostic delay, especially if autoimmune phenomena are the first manifestations of primary immunodeficiency [2]. Autoimmune phenomena might occur as the first symptom in 30% of patients with primary antibody deficiencies [3]. In a recent study focused on noninfectious CVID complications, autoimmune thrombocytopenic purpura (AITP) was most common (16.2%), followed by autoimmune hemolytic anemia (AIH 7.7%), amongst the 632 patients followed since 1974. Other associated autoimmune conditions include rheumatoid arthritis (2.7%) and uveitis (1%). Rarer autoimmune complications are psoriasis, psoriatic arthritis, vitiligo, alopecia, autoimmune thyroiditis, antiphospholipid syndrome, Sjogren syndrome, vasculitis, type 1 diabetes, myasthenia gravis, autoimmune pancreatitis, and severe oral ulcers [4]. Polyclonal lymphadenopathy occurred in 20–40% of patients [4]. Clinically, this often presents as generalized lymphadenopathy and splenomegaly. The challenge is to differentiate it from lymphoma [5]. In 20% of patients with a CVID-like phenotype, monogenic defects responsible for immune deregulation have been identified. Examples include CTLA4 and LRBA deficiency [6,7], nuclear factor kB (NFkB) mutations [8,9] and mutations of the catalytic subunit of phosphoinositide 3-kinase delta (PI3Kdeta) [10]. However, in the majority of patients with CVID, the pathogenesis of noninfectious phenomena is still unknown.

Similar symptoms were observed in systemic lupus erythematosus (SLE): cytopenia, generalized lymphadenopathy, hepatomegaly, splenomegaly, and interstitial lung disease. The clinical symptoms of CVID and systemic rheumatic diseases overlap. Evidence has accumulated that the coincidence of primary immunodeficiency (PID) and autoimmune diseases is high [11,12,13]. A secondary immunodeficiency-like state is present in a significant number of rheumatologic patients. Low serum levels of main immunoglobulins (Ig) and subclasses IgG3 and IgG4 are frequent, although in many cases laboratory abnormalities are not related to increased susceptibility to infections [14]. A recent study of patients with rheumatic diseases identified genetic variants that are responsible for PID in participants who developed hypogammaglobulinemia [11].

Immunophenotyping of the B cell compartment in the peripheral blood is a routine evaluation in patients with primary hypogammaglobulinemia. In CVID, detailed findings of B cell maturation can be classified according to several systems, of which two of the most popular are Freiburg [15] and EUROclass [16]. Characterization of CD19+ B cell subsets in CVID is classified according to low Ig switched memory (CD19+ CD27+ IgM− IgD−), B cell (smB) proportions, and abnormally high proportions of CD21^low^ B cells. In addition to these two cell subsets, the EUROclass classification also uses an abnormal expansion of transitional B cells (CD19+ CD27− CD38+) for further subgrouping [16].

Low smB cell subsets are an abnormality present in 80% of patients with CVID; however, it is not specific for CVID. More detailed studies that assessed the correlation between B cell maturation and the phenotype of CVID have produced mixed results. In some studies, diminished smB cell count [17], reduced naïve B cells [18], and expansion of B cells with reduced CD21 expression (CD21^low^ B cells) correlated with autoimmune phenomena or splenomegaly [16,19].

Although CVID is a disease of defective B cell maturation, various reports have associated CVID with T cell compartment abnormalities, such as CD4+ T cell lymphopenia with reduced subset counts of naive CD4+ T cells [20] and naive CD8+ T cells [21]. A reduced percentage of naïve CD4+ T cells was associated with complications and poor prognosis in CVID [22].

Regulatory T cells (Tregs), T helper 17 (Th17), and follicular T helper 17 (Tfh17) cells are reduced in patients with complicated CVID phenotypes [18]. T cells in patients with CVID have lower proliferative capacities [23] and abnormal cytokine production [24]. Recent studies have shown the involvement of follicular T cells in CVID pathogenesis [25] An increase in the circulating memory CD4+ T cells of CVID patients with noninfectious complications has been reported [26].

In contrast to PID, immunophenotyping of B and T cells in SLE and other autoimmune diseases is mainly used in scientific research and clinical trials [27]. Therefore, physicians are not familiar with the interpretation and utility of lymphocyte subset counts in clinical practice. The data showed that IgM memory B cells were significantly decreased in patients with SLE. In contrast, transitional B cells were significantly increased in SLE and other autoimmune disorders [28]. The population of plasmablasts also increased in active SLE [29].

Until now, studies comparing B and T cell subsets from patients with PID and patients with rheumatic diseases are limited. In one study, patients with primary and secondary hypogammaglobulinemia in the course of different rheumatic diseases were observed [30]. Another study involved the analysis of polymyalgia rheumatica patients treated with systemic glucocorticoids [31]. Both studies aimed to identify the distinction between primary and secondary hypogammaglobulinemia.

We analyzed the maturation of B and T lymphocytes in the peripheral blood of patients with CVID who were divided into two groups: patients with a phenotype limited to infections (CVID-OI) and patients with noninfectious, autoimmune complications (CVID-C). These results were compared with those of patients diagnosed with SLE and healthy controls (HCs). The aim of this study was to identify immunological markers of autoimmune phenomena associated with CVID.

## 2. Materials and Methods

### 2.1. Patients

The study population was selected from consecutive adult patients (≥18 years old) who were under the care of the outpatient clinic of the Department of Internal Medicine, Pulmonology, Allergy, and Clinical Immunology, Central Clinical Hospital of the Ministry of National Defense, Military Institute of Medicine in Warsaw, Poland, between January 2016 and December 2019.

The study participants included confirmed CVID patients diagnosed according to the European Society for Immunodeficiencies’ criteria [32] and were treated under the Polish Ministry of Health’s drug programs, B.62 and B.78. CVID clinical phenotypes were defined according to the literature [32]. Group 1, or CVID-C (*n* = 24), included patients with CVID who suffered from increased susceptibility to infections and at least one other clinical event beyond increased susceptibility to infections attributable to PID [33]. Group 2, or CVID-OI (*n* = 9), included patients with only the infectious phenotype. Group 3 included patients with SLE (*n* = 17) who fulfilled the Systemic Lupus International Collaborating Clinics’ (SLICCs’) criteria [34] and had no clinical signs of immunodeficiency.

Healthy controls (HC) were selected from age-matched volunteers from hospital employees without any signs, symptoms, or history of immunodeficiency and/or autoimmunity.

### 2.2. Compliance with Research Ethics Standards

The study protocol was approved by the Bioethics Committee of the Military Institute of Medicine (approval no. 7/WIM/2020). All patients were informed in detail orally about the course, aims, and scope of this research. Blood sampling was limited to routine assessments. Separate written consent for blood sampling and review of records were not required by the IRB due to the retrospective nature of this study. All patient data were confidential, and the study procedures complied with the Declaration of Helsinki.

### 2.3. Flow Cytometry Analysis

All blood samples were drawn during routine visits. If the CVID patients were on immunoglobulin replacement therapy (IgRT), blood samples were drawn before the day of IgG infusion according to the national regulations for treatment reimbursement.

Lymphocyte subset percentages were determined according to literature [15,35] by flow cytometry using a panel of monoclonal antibodies using FACS Canto II BD flow cytometry (Becton Dickinson (BD) Biosciences, Franklin Lakes, NJ, USA). Subsequently, all eight-color surface staining panels for the basic subpopulation of lymphocytes were evaluated as follows: CD4 FITC, CD3 PerCP-Cy5-5, CD19 PE-Cy7, CD8 APC, CD16 APC-H7, and CD45 V500 (BD Biosciences).

B cell subpopulations were defined using the following antibodies: IgD PE, CD27 PerCP-Cy5-5, CD19 PE-Cy7, IgM APC, CD38 APC-H7, CD21 V450, and CD45 V500 (BD Biosciences).

CD4 T cell and CD8 maturation were defined using CD4 FITC, CD196 PE, CD197 PerCP-Cy5-5, CD45RO PE-Cy7, CD45RA APC, CD3 APC-H7, CD8 V450, and CD45 V500 (BD Biosciences) antibodies.

For Th17 cells, CD4 FITC, CD196 PE, CD45RO PE-Cy7, and CD45 V500 (BD Biosciences) antibodies were used.

Recent thymic emigrant (RTE) CD4+ or CD8+ cells were analyzed using CD4 FITC, CD62L PE, CD31 PerCP-Cy5-5, CD45RO PE-Cy7, CD45 RA APC, CD3 APC-H7, and CD8 V450 CD45 V500 (BD Biosciences) antibodies. 

For Tregs, we used CD127 FITC, CD4 PerCP-Cy5-5, CD25 APC, CD3 APC-H7, CD45 V500 (BD Biosciences) antibodies, and for regulatory B cells (Bregs), CD1d PE, CD19 PE-Cy7, CD5 APC, and CD45 V500 (BD Biosciences) antibodies were used.

After surface staining for 15 min at 21 °C in the dark, erythrocytes were lysed with 2 mL of BD Pharm Lyse buffer (BD Biosciences) for 10 min. Following centrifugation and washing with Cell Wash buffer (BD Biosciences), the mixture was stored in the dark for analysis by flow cytometry within 2 h. Data were analyzed with DIVA Analysis software (version 8.0.1, BD Biosciences, San Jose, CA 95131 USA) and Infinicyt 1.8 Flow Cytometry (Cytognos, Salamanca, Spain).

Lymphocyte counts were obtained using a SYSMEX XN-1500 (Sysmex Corp., Kobe, Japan) hematological analyzer.

Internal quality control was performed daily by checking the optical detector, aligning lasers, and fluid systems using CS&T IVD Beads BD FACS Diva (BD Biosciences), San Jose, CA 95131 USA, respectively, according to the manufacturers’ guidelines. Internal reference values of lymphocyte counts and proportions are presented in Supplementary Materials (Tables S1–S3).

We distinguished the following subpopulations in B cell maturation:

- transitional B cells: IgM++ IgD++ CD38++ CD27− CD19+ CD45+
- naïve B cells: IgM+ IgD++ CD38+ CD27− CD19+ CD45+
- nonswitched memory B cells (marginal zone-like B cells): IgM++ IgD+ CD38+ CD27+ CD19+ CD45+
- class-switched memory B cells: IgM− IgD− CD38+ CD27+ CD19+ CD45+
- plasmablasts: IgM−/+ IgD− CD38+++ CD27++ CD19+ CD45+
- CD21^low^ B cells: IgM+ IgD+ CD38+low CD27− CD21+low CD19+ CD45+

We distinguished the following subpopulations in T cell CD4+ or CD8+ maturation:

- RTE T cells: CD45RA+ CD62L+ CD31+ CD3+ CD45+
- naïve T cells: CD45RA+ CD197+ CD3+ CD45+
- effector T cells: CD45RA+ CD197− CD3+ CD45+
- central memory T cells: CD45RO+ CD197+ CD3+ CD45+
- effector memory T cells: CD45RO+ CD197− CD3+ CD45+
- RTE T cells: CD45RA+ CD62L+ CD31+

We distinguished the following other lymphocytes subpopulations:

- Bregs: CD19+ CD5+ CD1d^high^
- Tregs: CD3+ CD4+ CD25^high^ FoxP3+ CD127−
- Th17: CD3+ CD4+ CD45RO+ CD196+

Representative B and T lymphocyte maturation gating strategies in patients are presented in Figures S1–S3 (Supplementary Materials).

### 2.4. Statistical Analysis

All statistical analyses were performed using Statistica^®^ software (version 13.0; TIBCO Software, Palo Alto, CA, USA). Statistical significance was set at *p* < 0.05. The results are expressed as medians (Q1–Q3) of the lymphocyte populations. For group comparisons, the Kruskal–Wallis, analysis of variance (ANOVA), and post hoc analysis tests were used.

## 3. Results

### 3.1. The Clinical Characteristics of Patients

Among the 33 patients with CVID, 14 were women and 19 were men, and the overall mean age at the time of blood sampling was 37.75 years (min–max: 21–66 years). Among the 9 patients with CVID-OI, 4 (44%) were men, and among 24 patients with CVID-C, 16 (66%) were men. The mean age was 37.0 years (±17.2) and 38.4 years (±12.4) in patients with CVID-OI and CVID-C, respectively. By the time of blood sampling, two patients were receiving low-dose prednisolone (5 mg/day), and one was being treated with methotrexate and etanercept due to psoriatic arthritis.

All CVID patients had a positive history of increased susceptibility to infections; however, only 9 of 33 (27%) presented with a clinical phenotype limited to infections. The clinical characteristics of the patients are summarized in Table 1. All 17 SLE patients were female, mean age 43.05 years (min–max 18–60). The SLE patients had low or mild disease activity according to the Systemic Lupus Erythematosus Disease Activity Index 2000 SLEDAI2K (mean value 3.4). Sixteen patients with SLE were treated with antimalarials, and ten patients received prednisolone (5–15 mg/day).

### 3.2. Peripheral Main Lymphocyte Subsets, Tregs, Bregs, and Th17 Cells

The proportion of total lymphocytes was low in the CVID-C and SLE groups. It differed significantly from that of the HC group. CVID-OI patients had all lymphocyte proportions similar to those of HC.

The proportion of T lymphocytes was the lowest in CVID-C and differed significantly from that in HCs. In SLE patients, T lymphocytes were also significantly lower than in the HC group. The proportion of T lymphocytes in CVID-OI was similar to that in HC. The results are summarized in Table 2.

Among the T lymphocytes, the proportion of CD4+ T cells was significantly reduced in patients with CVID-C, CVID-OI, and SLE compared to that in HC. There were no differences in the proportion of CD8+ T cells between the disease groups and HC. The CD4/CD8 ratio was reduced in CVID-C and CVID-OI compared with that in HCs.

The proportion of circulating B cells was reduced in CVID-C and differed significantly from that in HC and SLE.

The NK cell proportion was marked decreased in CVID-C patients compared to HC patients.

**Figure 1:**
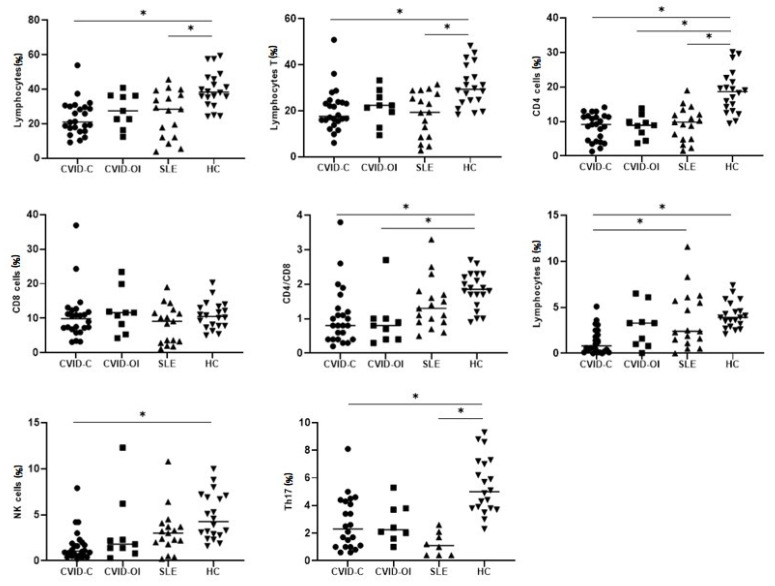
Differences in the median percentages of lymphocyte subpopulations between the CVID with complicated phenotype (CVID-C) group, the CVID phenotype limited to only infections (CVID-OI), patients with systemic lupus erythematosus (SLE), and healthy controls (HCs). Data expressed as median (Q1–Q3), * *p* < 0.05.

The proportion of Th17 cells was reduced in CVID-C and SLE and differed significantly from that in HCs. The Th17 cell counts were the lowest in SLE (Figure 1).

**Figure 2:**
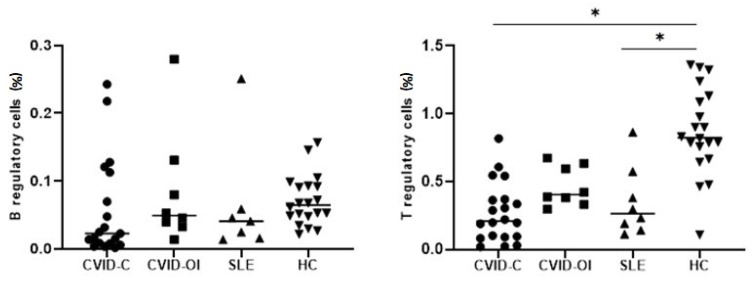
Differences in the median percentages of T regulatory cells (Tregs) and B regulatory cells (Bregs) between the CVID with complicated phenotype (CVID-C) group, CVID phenotype limited to only infections (CVID-OI group), patients with systemic lupus erythematosus (SLE), and healthy controls (HCs). Data are expressed as median (Q1–Q3) * *p* < 0.05.

Treg counts were low in CVID-C and SLE patients and differed significantly from HCs. Breg counts were lowest in CVID-C patients, but no significant differences were noted between the disease groups and the HC group (Figure 2). Treg counts were low in CVID-C and SLE patients and differed significantly from HCs.

Considering the absolute numbers of the above-mentioned lymphocyte populations, the same trends were observed. A difference was observed only in the CD8 lymphocytes of SLE patients, which were significantly less than in the HCs (Table 2).

### 3.3. B Lymphocyte Maturation

**Figure 3:**
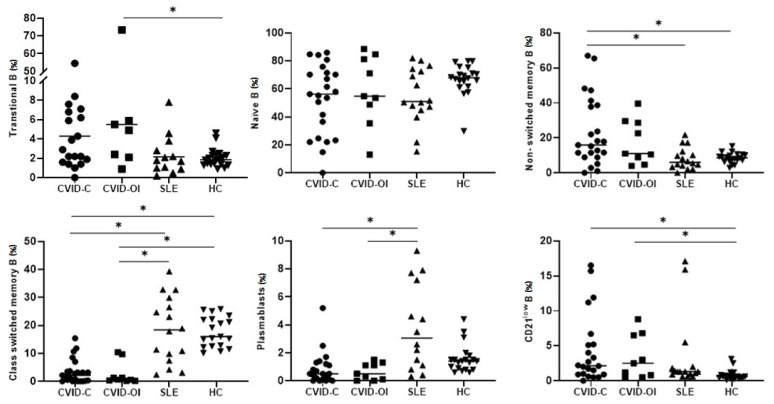
Differences in the proportions of B lymphocyte maturation between the CVID with complicated phenotype (CVID-C) group, CVID phenotype limited to only infections (CVID-OI group), patients with systemic lupus erythematosus (SLE), and healthy controls (HCs). Data expressed as median (Q1–Q3), * *p* < 0.05.

**Figure 4:**
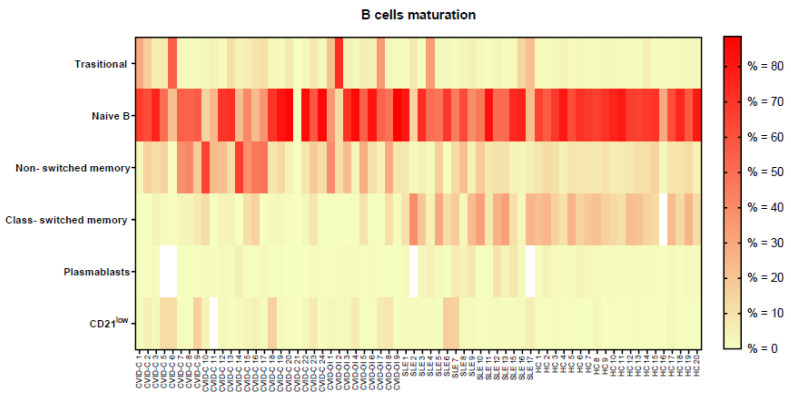
The proportions of B lymphocyte maturation for each patient with CVID with complicated phenotype (CVID-C) group, CVID phenotype limited to only infections (CVID-OI group), patients with SLE, and healthy controls (HCs). Data expressed as a median percentage (%).

Analysis of the maturation of B-lymphocytes showed that the proportion of transitional B cells was highest in CVID-OI and differed significantly from that in HCs. The frequency of nonswitched memory B cells was the highest in patients with CVID-C. The difference in nonswitched memory B cell counts was significant between the CVID-C and SLE groups and between the CVID-C and HC groups. Class-switched memory B cell percentages were low in CVID-C and CVID-OI. The difference was significant in comparison with the percentage of smB cells in HCs and SLE. The proportion of CD21^low^ B cells was higher in CVID-C and CVID-OI than in HCs. Plasmablasts were significantly higher in SLE patients than in CVID-C and CVID-OI patients (Table 3, Figure 3). The proportions of B lymphocyte maturation for each patient with CVID-C, CVID-OI, SLE, and HC are presented on heat maps in Figure 4.

### 3.4. T Lymphocyte Maturation

**Figure 5:**
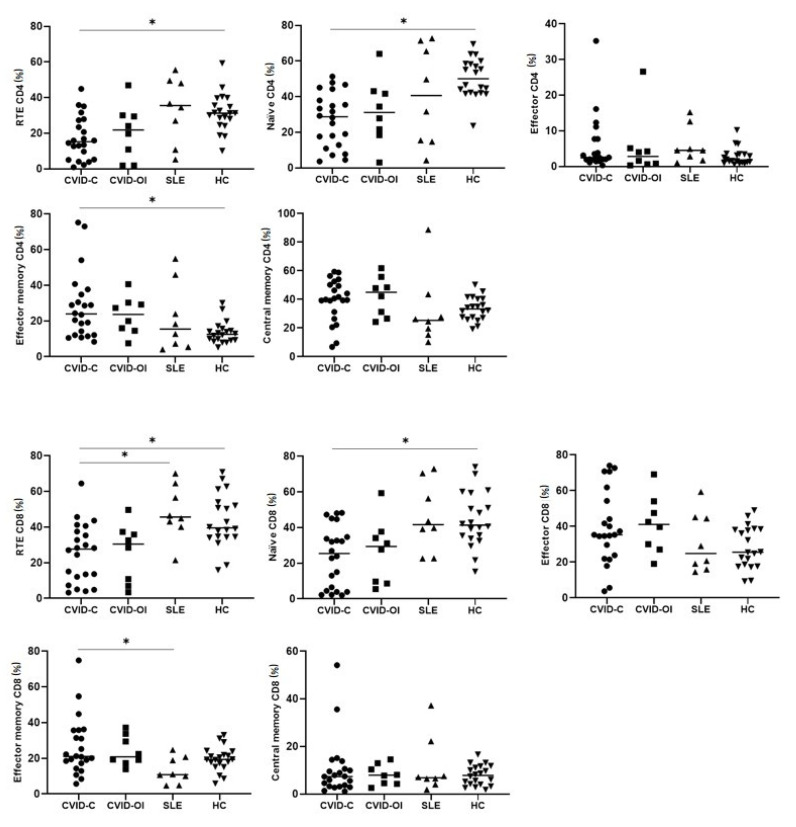
Differences in the proportions of T lymphocyte maturation between CVID with complicated phenotype (CVID-C) group, CVID phenotype limited to only infections (CVID-OI group), patients with SLE, and healthy controls (HCs). Data expressed as median (Q1–Q3), * *p* < 0.05.

**Figure 6:**
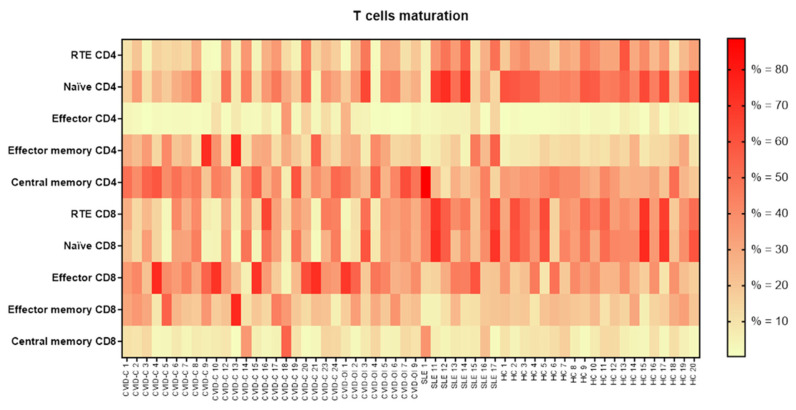
The proportions of T lymphocyte maturation for each patient with CVID with complicated phenotype (CVID-C) group, CVID phenotype limited to only infections (CVID-OI group), patients with systemic lupus erythematosus (SLE), and healthy controls (HCs). Data expressed as a median percentage (%).

To examine the abnormalities in T cell maturation, we delineated CD4+ and CD8+ cells. RTE CD4+ and naïve CD4+ T cell percentages were significantly reduced in CVID-C patients compared to that in HCs. The proportion of effector memory CD45RO+ CD197-CD4+ T cells significantly increased in CVID-C compared to the HCs. The RTE CD8+ T cell proportion was low in CVID-C and differed significantly from SLE (the highest proportion) and HCs. Naïve CD8+ T cell counts were significantly reduced in patients with CVID-C compared to HCs. The proportion of effector memory CD8+ T cells was similar in the CVID and HC groups. The proportion of effector memory CD8+ T cells was the lowest in the SLE group and significantly differed from CVID-C (Table 4, Figure 5). The proportions for the T lymphocyte maturation of each patient with CVID-C, CVID-OI, and SLE and HCs are presented on heat maps (Figure 6).

## 4. Discussion

Using flow cytometry, we determined the lymphocyte profiles of four patient groups: CVID-C, CVID-OI, SLE, and HCs.

In our study, patients with a complicated CVID phenotype had low proportions of Tregs and NK cells. Decreased proportions of Tregs in CVID were first reported by Fevang et al. [36]. They included 26 patients diagnosed with CVID according to the WHO classification. In CVID patients, they found significantly reduced expression of the transcription factor FoxP3 in CD3+ cells and a decreased proportion of T CD4+CD25^high^FoxP3+ cells in the CD4(+) population, as measured by flow cytometry. The lowest proportion of Tregs was found in patients with CVID and splenomegaly (spleen size > 13 cm on ultrasound examination). Treg proportions correlated negatively with neopterin levels as a marker of chronic inflammation [36]. Kofod-Olsen et al. in a study of 26 patients with CVID, demonstrated an association between decreased levels of Tregs and autoimmune phenomena [37]. Moreover, in patients with CVID, the functions of Tregs were disturbed [36,38]. One study that included 20 children with CVID (mean age 173 months) found no difference in Tregs between CVID cases and healthy controls [39]. This suggests different etiopathologies of childhood vs. adult-onset CVID.

We found low NK cells in the CVID-C group (median, 54 cells/μL). Similarly, patients with CVID from the French registry who had a severe reduction of NK cells (<50/μL at study inclusion) presented with a complicated phenotype [40]. Therefore, our results support the finding that low NK cell numbers can be a biomarker of complicated CVILD [41,42].

We found that patients with CVID-C had reduced Th17 proportions. Th17 cell involvement in CVID has not been well studied [18,43]. Barbosa et al. were the first to evaluate circulating Th17 cells in 30 patients with CVID [43]. They found a significant reduction in Th17 cells in CVID patients who had less than 2% smB cells and more than 10% of cd21low B cells. This suggested that there is a link between B cell maturation disturbances and maintenance of Th17 cells [43]. Edwards et al. found low Th17 numbers in patients with predominantly deficient antibodies and noninfectious complications [18].

Bregs play a critical role in immune homeostasis and tolerance. Despite extensive efforts to phenotypically characterize Bregs, we still lack a definitive set of phenotypic markers or a signature transcriptional regulator (equivalent to FoxP3 Tregs) that enables us to comprehensibly identify Bregs [44]. In our study, Bregs were identified as CD19+ CD5+ CD1d^high^. CD1d is a major phenotypic marker highly expressed in many Breg cells, and it may play a crucial role in Breg-cell-mediated suppression [45]. The upregulation of CD1d on B cells is associated with B-cell-mediated protection against inflammation [44,46]. In our study, we observed a tendency for decreased Bregs in CVID-C patients; however, this was not statistically significant. Yesillic et al. found significantly lower proportions of Breg cells in 25 adult patients with CVID [47]. On the other hand, Kofod-Olsen et al. showed that an elevated frequency of pro-B10 cells in CVID correlated with autoimmunity and splenomegaly [37]. In another study that included 42 adult patients with CVID, the Breg number was low, and their function was disturbed [48]. Although 40% of patients in that study had autoimmune symptoms, there was no correlation between the reduction of Breg cells and autoimmunity. Discrepancies among the studies might be a result of different methodologies of Breg assessment and differences in the incidence and types of autoimmunity in published cohorts.

T cell subsets in our CVID-C patients were characterized by low naive CD4+T cells, low RTE CD4+ T cells, low naïve CD8+T cells, and low RTE CD8+T cells. These results illustrate that profound T cell pool abnormalities are part of the picture of complicated CVID. Signs of senescence in T cell maturation have been reported in CVID-C patients. Stuchlý et al. hypothesized that in CVID with autoimmune thrombocytopenia, naive CD4+ T cell pool depletion occurred and increased the likelihood of promoting autoreactive T cells to memory stages concurrently with B cell activation [49]. Activated B cells, which cannot progress through germinal center reactions, fail to produce isotype-switched antibodies [50].

To better understand autoimmunity, we compared the cytometry results between CVID and SLE patients, as a prototypic autoimmune disease. Patients with CVID and SLE both had lymphopenia. We found that only a more detailed analysis of peripheral lymphocyte subset counts showed differences; specifically, smB cells and plasmablasts were reduced in CVID, which is in agreement with other studies [30]. Therefore, low smB cells detected in patients with autoimmunity should increase the awareness of immunodeficiency. In a recently published study, retrospective evaluation of available peripheral lymphocyte subset counts revealed lower proportions of class-switched memory B cells [9] in patients with inflammatory rheumatic diseases. These patients also presented with mutations in PID genes and were subsequently revealed to have hypogammaglobulinemia.

The expansion of the CD21^low^ B cell compartment and increased plasmablasts are expected findings in autoimmune diseases. This was not the case in the SLE patients in our study. Moreover, they had the lowest Th17 percentages. This can be explained by the low disease activity in our patients [50]. However, Treg imbalance persists despite low SLE activity and might be a further attractive therapeutic goal [51].

Our study has some limitations. First, the sample size of each group was relatively small. Due to the limited sample size, we were not able to assess the relationships between specific clinical symptoms and lymphocyte subsets. However, the limited number of CVID patients is to be expected as it is a rare disease, and our sample size is similar to that of other single-center studies. On the other hand, we collected extensive data from each patient and only included patients with a confirmed diagnosis.

In conclusion, we confirmed that patients with CVID and autoimmune phenomena had reduced Treg, Th17, and NK cells.

Future studies are needed to confirm whether reductions of Treg, Th17, and NK cells might be a biomarker of more complicated CVID cases. Nevertheless, class-switched memory B cells can help distinguish patients with different causes of autoimmunity. Our results suggest that T and B cell maturation analyses should be performed routinely in clinical practice.

## Figures and Tables

**Table 1:** The clinical characteristics of patients with CVID and SLE.

Clinical Phenotypes and Organ Complications in CVID Patients (*n* = 33)	
No disease-related complications	9 (27%)
Bronchiectasis	4 (12%)
Splenomegaly	7 (21%)
Autoimmunity	20 (60%)
Thrombocytopenia	10 (33%)
Hemolytic anemia	6 (18%)
Addison–Biermer disease	2 (6%)
Vitiligo	1 (3%)
Chronic seronegative polyarthritis	2 (6%)
Alopecia areata	1 (3%)
Nonspecific inflammatory bowel disease	1 (3%)
Psoriasis	3 (6%)
Polyclonal lymphocytic infiltration	
Generalized lymphadenopathy	19 (57%)
Granulomatous lesions (histopathological confirmation)	9 (27%)
Immunoglobulin replacement therapy	29
Immunoglobulin naïve	3
Prednisolone	2; dose 5 mg/day
Methotrexate and etanercept	1
Rituximab in anamnesis	2
**Clinical data of SLE patients (*n* = 17)**	
SLEDAI2K	3.6 (min 0–max 9)
Treatment	16/17
Prednisolone	10 (58%); dose: 9 mg/day (min 5–max 15 mg)
Antimalarials	16 (94%)
Immunosuppressive medication	
Methotrexate	2 (11%)
Rituximab in anamnesis	1 (5%)

CVID: Common variable immunodeficiency, SLE: systemic lupus erythematosus, SLEDAI2K: Systemic Lupus Erythematosus Disease Activity Index 2000.

**Table 2:** Differences in the median of lymphocyte subpopulations and absolute numbers between (a) the CVID with complicated phenotype (CVID-C) group, (b) CVID phenotype limited to only infections (CVID-OI group), (c) patients with systemic lupus erythematosus (SLE), and (d) healthy controls (HCs). Data expressed as median (Q1–Q3). * *p* < 0.05. ANOVA: analysis of variance, Post Hoc: post hoc analysis tests.

As Median (Q1–Q3)	CVID-C (a) *n* = 24	CVID-OI (b) *n* = 9	SLE (c) *n* = 17	HC (d) *n* = 20	*p* < 0.05 Group a-b-c-d ANOVA, Kruskal–Wallis	*p* < 0.05 between Groups Post Hoc Test
% of all cells						
Lymphocytes	21.0 (16.6–29.8)	27.5 (22.7–36.3)	28.4 (12.1–36.6)	38.3 (33.2–46.3)	*p* = 0.0002	*a-d, *c-d
Lymphocytes T	17.6 (15.7–23.7)	22.4 (19.5–25.9)	19.3 (8.7–27.4)	29.5 (24.0–37.2)	*p* = 0.0011	*a–d, *c–d
CD4 cells	9.1 (4.3–11.4)	8.9 (6.8–9.9)	9.8 (4.9–12.0)	18.6 (13.6–22.0)	*p* < 0.0001	*a–d, *b–d, *c–d
CD8 cells	9.8 (7.0–12.0)	11.6 (8.3–11.9)	9.1 (3.3–11.5)	10.5 (7.8–13.2)	-	-
Lymphocytes B	0.8 (0.1–2.2)	3.3 (1.0–3.4)	2.4 (1.5–5.7)	3.9 (3.0–5.0)	*p* < 0.0001	*a–c, *a–d
NK cells	1.0 (0.5–1.9)	1.8 (1.4–2.3)	3.0 (2.0–3.7)	4.2 (2.8–7.0)	*p* = 0.0001	*a–d
Bregs	0.023 (0.008–0.113	0.050 (0.037–0.106)	0.041 (0.016–0.059)	0.065 (0.049–0.093)	-	-
Tregs	0.209 (0.093–0.366)	0.404 (0.356–0.613)	0.265 (0.167–0.478)	0.824 (0.711–1.109)	*p* < 0.0001	*a–d, *c–d
Th17	2.3 (1.0–4.3)	2.2 (1.8–3.7)	1.1 (0.4–1.9)	5.0 (3.8–7.1)	*p* < 0.0001	*a–d, *c–d
(cells/µL)						
WBC	5575 (4605–7555)	6600 (5440–7370)	5690 (3630–8310)	6555 (4930–7535)	-	-
Lymphocytes	1201 (755–2145)	1986 (1119–2402)	1115 (1005–1576)	2037 (1838–2934)	*p* = 0.0002	*a–d, *c–d
Lymphocytes T	1071 (701–1614)	1457 (961–2093)	887 (570–1125)	1660 (1409–2292)	*p* = 0.0004	*a–d, *c–d
CD4 cells	458 (305–553)	574 (372–680)	418 (288–499)	978 (756–1559)	*p* < 0.0001	*a–d, *c–d
CD8 cells	580 (305–809)	814 (374–1089)	319 (162–582)	624 (457–791)	*p* = 0.0269	*c–d
Ratio CD4/CD8	0.8 (0.4–1.2)	0.8 (0.4–1.0)	1.3 (0.9–1.7)	1.8 (1.5–2.5)	*p* = 0.0003	*a–d, *b–d
Lymphocytes B	47 (12–127)	212 (117–332)	145 (69–222)	216 (190–284)	*p* < 0.0001	*a–d
NK cells	54 (32–100)	152 (96–488)	126 (87–234)	245 (204–447)	*p* = 0.0001	*a–d
Bregs	1 (0–4)	4 (2–9)	2 (1–3)	4 (3–7)	-	-
Tregs	15 (4–21)	26 (22–30)	20 (12–28)	55 (37–82)	*p* < 0.0001	*a–d, *c–d
Th17	130 (73–190)	131 (94–284)	77 (35–113)	256 (209–494)	*p* < 0.0001	*a–d, *c–d

**Table 3:** Differences in the proportions of B lymphocyte maturation between (a) the CVID with complicated phenotype (CVID-C) group, (b) CVID phenotype limited to only infections (CVID-OI group), (c) patients with systemic lupus erythematosus (SLE), and (d) healthy controls (HCs). Data expressed as median (Q1–Q3). * *p* < 0.05. ANOVA: analysis of variance, Post Hoc: post hoc analysis tests.

As Median (Q1–Q3)	CVID-C (a) *n* = 24	CVID-OI (b) *n* = 9	SLE (c) *n* = 17	HC (d) *n* = 20	*p* < 0.05 Group a-b-c-d ANOVA, Kruskal–Wallis	*p* < 0.05 between Groups Post Hoc Test
% of B cells						
Transitional B	4.3 (1.9–8.4)	5.5 (2.4–21.6)	2.1 (1.0–6.2)	1.8 (1.4–2.3)	*p* = 0.0149	*b–d
Naïve B	56.3 (24.6–71.5)	54.8 (48.8–81.3)	51.0 (46.0–73.4)	68.0 (63.5–73.1)	-	-
Nonswitched memory	15.9 (8.9–38.7)	11.0 (8.8–28.7)	6.0 (3.7–11.1)	8.6 (6.9–10.3)	*p* = 0.0036	*a–c, *a–d
Class-switched memory	2.2 (0.2–3.7)	0.6 (0.3–1.3)	18.4 (8.6–28.2)	17.6 (12.7–22.8)	*p* < 0.0001	*a–c, *a–d, *b–c, *b–d
Plasmablasts	0.5 (0.1–1.4)	0.5 (0.1–1.1)	3.9 (1.3–7.8)	1.4 (0.8–1.6)	*p* = 0.0004	*a–c, *b–c
CD21^low^ B cells	2.2 (0.9–6.7)	2.5 (0.8–6.5)	1.3 (0.9–1.9)	0.6 (0.4–0.9)	*p* = 0.0005	*a–d, *b–d
Transitional B	4.3 (1.9–8.4)	5.5 (2.4–21.6)	2.1 (1.0–6.2)	1.8 (1.4–2.3)	*p* = 0.0149	*b–d
Naïve B	56.3 (24.6–71.5)	54.8 (48.8–81.3)	51.0 (46.0–73.4)	68.0 (63.5–73.1)	-	-
Nonswitched memory	15.9 (8.9–38.7)	11.0 (8.8–28.7)	6.0 (3.7–11.1)	8.6 (6.9–10.3)	*p* = 0.0036	*a–c, *a–d

**Table 4:** Differences in the proportions of T lymphocytes maturation between (a) the CVID with complicated phenotype (CVID-C) group, (b) CVID phenotype limited to only infections (CVID-OI group), (c) patients with systemic lupus erythematosus (SLE), and (d) healthy controls (HCs). Data expressed as median (Q1–Q3), * *p* < 0.05. ANOVA: analysis of variance, Post Hoc: post hoc analysis tests.

As Median (Q1–Q3)	CVID-C (a) *n* = 24	CVID-OI (b) *n* = 9	SLE (c) *n* = 17	HC (d) *n* = 20	*p* < 0.05 Group a-b-c-d ANOVA, Kruskal–Wallis	*p* < 0.05 between Groups Post Hoc Test
% of CD4 cells						
Recent thymic emigrants (RTE) CD4	15.2 (5.2–27.3)	21.8 (6.3–29.7)	35.5 (18.5–48.7)	31.2 (26.3–37.6)	*p* = 0.0031	*a–d
Naïve CD4	28.7 (12.8–37.9)	31.1 (20.0–42.3)	40.6 (15.0–68.4)	50.0 (42.1–58.3)	*p* = 0.0009	*a–d
Effector CD4	2.4 (1.9–7.7)	2.8 (0.7–4.6)	4.5 (2.2–8.7)	1.8 (1.1–3.4)	-	-
Effector memory CD4	23.9 (12.1–34.8)	23.6 (15.2–29.7)	15.4 (6.3–34.9)	12.5 (9.2–15.0)	*p* = 0.0126	*a–d
Central memory CD4	40.2 (31.2–50.2)	44.9 (28.8–52.0)	25.3 (17.4–35.5)	33.2 (27.2–40.3)	-	-
CD21^low^ B cells	2.2 (0.9–6.7)	2.5 (0.8–6.5)	1.3 (0.9–1.9)	0.6 (0.4–0.9)	*p* = 0.0005	*a–d, *b–d
Transitional B	4.3 (1.9–8.4)	5.5 (2.4–21.6)	2.1 (1.0–6.2)	1.8 (1.4–2.3)	*p* = 0.0149	*b–d
Naïve B	56.3 (24.6–71.5)	54.8 (48.8–81.3)	51.0 (46.0–73.4)	68.0 (63.5–73.1)	-	-
Nonswitched memory	15.9 (8.9–38.7)	11.0 (8.8–28.7)	6.0 (3.7–11.1)	8.6 (6.9–10.3)	*p* = 0.0036	*a–c, *a–d
% of CD8 cells						
Recent thymic emigrants (RTE) CD8	27.6 (12.1–37.5)	30.4 (8.9–36.5)	45.6 (41.6–60.4)	39.5 (34.4–52.9)	*p* = 0.0006	*a–c, *a–d
Naïve CD8	25.4 (4.5–34.7)	29.3 (9.1–35.9)	41.6 (30.9–63.3)	41.3 (34.6–55.2)	*p* = 0.0019	*a–d
Effector CD8	35.3 (23.8–54.3)	41.1 (28.5–50.7)	24.8 (17.5–44.7)	25.5 (18.1–38.2)	-	-
Effector memory CD8	21.0 (17.2–35.7)	20.8 (18.2–31.5)	10.8 (7.4–19.8)	19.3 (16.2–22.9)	*p* = 0.0490	*a–c
Central memory CD8	7.3 (3.2–10.6)	7.9 (4.4–11.6)	6.8 (5.2–14.8)	7.8 (4.1–11.4)	-	-

## Data Availability

Data supporting reported results can be made available by request from the corresponding author.

## Supplementary Materials

The following are available online at https://www.mdpi.com/article/10.3390/jcm10153356/s1, Table S1: Internal laboratory reference values of lymphocyte subpopulations and absolute numbers, Table S2: Internal laboratory reference values if the proportions of B lymphocyte maturation, Table S3: Internal laboratory reference values if the proportions of T lymphocyte maturation, Figure S1: Representative gating strategy of main lymphocytes subpopulation and T regulatory (Tregs), B regulatory cells (B regs) and Th17 cells in study group, Figure S2: Representative B lymphocytes maturation gating strategy in study group. (A) B lymphocytes gating strategy: FSC-A vs. FSC-H plot: Gating the cells that have an equal area and height, thus removing clumps (greater FSC-A relative to FSC-H) and debris (very low FSC), CD45 vs. SSC-A plot: Selection of lymphocytes based on their SSC/CD45+ properties, CD19 vs. SSC-A plot: Selection of lymphocytes B based on their SSC/CD19+ properties. (B) Representative dot plots of each maturation B subsets cells: transitional B cells (orange), naïve B cells (blue), non-switched memory B cells (red), class switched memory B cells (yellow), CD21^low^ cells (purple) and plasmablasts (green) based on their IgD/IgM, IgD/CD38, CD21/CD38, IgM/CD38, CD27/CD21 properties (phenotypes of all cells described in section: material and method), Figure S3: Representative T lymphocytes maturation gating strategy in study group. (A) T lymphocytes gating strategy: FSC-A vs. FSC-H plot: Gating the cells that have an equal area and height, thus removing clumps (greater FSC-A relative to FSC-H) and debris (very low FSC), CD45 vs. SSC-A plot: Selection of lymphocytes based on their SSC/CD45+ properties, CD3 vs. SSC-A plot: Selection of lymphocytes T based on their SSC/CD3+ properties. CD4 vs. CD8 plot: Selection of lymphocytes T CD4+ (pink) and CD8+ (yellow) based on their CD4+ or CD8+ properties. (B) Representative dot plots of each maturation T CD4+ subsets cells: recent thymic emigrants T CD4+ cells, naïve CD4+ T cells, effector CD4+ T cells, central memory CD4+ T cells and effector memory CD4+ T cells based on CD197/CD45RO, CD62L/CD45RA, CD45RA/CD31 properties (phenotypes of all cells described in section: material and method). (C) Representative dot plots of each maturation T CD8+ subsets cells: recent thymic emigrants T CD8+ cells, naïve CD8+ T cells, effector CD8+ T cells, central memory CD8+ T cells and effector memory CD8+ T cells based on their CD197/CD45RO, CD62L/CD45RA, CD45RA/CD31 properties (phenotypes of all cells described in section: material and method).

## References

[1] Więsik-Szewczyk E., Jahnz-Różyk K. (2020). From Infections to Autoimmunity: Diagnostic Challenges in Common Variable Immunodeficiency. World J. Clin. Cases.

[2] Ziętkiewicz M., Więsik-Szewczyk E., Matyja-Bednarczyk A., Napiórkowska-Baran K., Zdrojewski Z., Jahnz-Różyk K. (2020). Shorter Diagnostic Delay in Polish Adult Patients With Common Variable Immunodeficiency and Symptom Onset After 1999. Front. Immunol..

[3] Bagheri Y., Vosughi A., Azizi G., Yazdani R., Kiaee F., Hafezi N., Alimorad S., Khoshmirsafa M., Seif F., Hassanpour G. (2019). Comparison of Clinical and Immunological Features and Mortality in Common Variable Immunodeficiency and Agammaglobulinemia Patients. Immunol. Lett..

[4] Ho H.E., Cunningham-Rundles C. (2020). Non-infectious Complications of Common Variable Immunodeficiency: Updated Clinical Spectrum, Sequelae, and Insights to Pathogenesis. Front. Immunol..

[5] Costagliola G., Consolini R. (2021). Lymphadenopathy at the crossroad between immunodeficiency and autoinflammation: An intriguing challenge. Clin. Exp. Immunol..

[6] Schubert D., Bode C., Kenefeck R., Hou T.Z., Wing J.B., Kennedy A., Bulashevska A., Petersen B.S., Schäffer A.A., Grüning B.A. (2014). Autosomal dominant immune dysregulation syndrome in humans with CTLA4 mutations. Nat. Med..

[7] Lo B., Zhang K., Lu W., Zheng L., Zhang Q., Kanellopoulou C., Zhang Y., Liu Z., Fritz J.M., Marsh R. (2015). Patients with LRBA deficiency show CTLA4 loss and immune dysregulation responsive to abatacept therapy. Science.

[8] Klemann C., Camacho-Ordonez N., Yang L., Eskandarian Z., Rojas-Restrepo J.L., Frede N., Bulashevska A., Heeg M., Al-Ddafari M.S., Premm J. (2019). Clinical and Immunological Phenotype of Patients With Primary Immunodeficiency Due to Damaging Mutations in NFKB2. Front. Immunol..

[9] Tuijnenburg P., Lango Allen H., Burns S.O., Greene D., Jansen M.H., Staples E., Stephens J., Carss K.J., Biasci D., Baxendale H. (2018). Loss-of-function nuclear factor κB subunit 1 (NFKB1) variants are the most common monogenic cause of common variable immunodeficiency in Europeans. J. Allergy Clin. Immunol..

[10] Elgizouli M., Lowe D.M., Speckmann C., Schubert D., Hülsdünker J., Eskandarian Z., Dudek A., Schmitt-Graeff A., Wanders J., Jørgensen S.F. (2016). Activating PI3Kδ mutations in a cohort of 669 patients with primary immunodeficiency. Clin. Exp. Immunol..

[11] Sogkas G., Dubrowinskaja N., Adriawan I.R., Anim M., Witte T., Schmidt R.E., Atschekzei F. (2020). High Frequency of Variants in Genes Associated with Primary Immunodeficiencies in Patients with Rheumatic Diseases with Secondary Hypogammaglobulinaemia. Ann. Rheum. Dis..

[12] Perazzio S.F., Granados Á., Salomão R., Silva N.P., Carneiro-Sampaio M., Andrade L.E.C. (2016). High Frequency of Immunodeficiency-like States in Systemic Lupus Erythematosus: A Cross-Sectional Study in 300 Consecutive Patients. Rheumatology.

[13] Errante P.R., Perazzio S.F., Frazão J.B., da Silva N.P., Andrade L.E.C. (2016). Primary Immunodeficiency Association with Systemic Lupus Erythematosus: Review of Literature and Lessons Learned by the Rheumatology Division of a Tertiary University Hospital at São Paulo, Brazil. Rev. Bras. Reumatol..

[14] Almaghlouth I., Su J., Johnson S.R., Pullenayegum E., Gladman D., Urowitz M. (2020). Acquired Low Immunoglobulin Levels and Risk of Clinically Relevant Infection in Adult Patients with Systemic Lupus Erythematosus: A Cohort Study. Rheumatol. Oxf. Engl..

[15] Warnatz K., Denz A., Dräger R., Braun M., Groth C., Wolff-Vorbeck G., Eibel H., Schlesier M., Peter H.H. (2002). Severe Deficiency of Switched Memory B Cells (CD27(+)IgM(-)IgD(−)) in Subgroups of Patients with Common Variable Immunodeficiency: A New Approach to Classify a Heterogeneous Disease. Blood.

[16] Wehr C., Kivioja T., Schmitt C., Ferry B., Witte T., Eren E., Vlkova M., Hernandez M., Detkova D., Bos P.R. (2008). The EUROclass Trial: Defining Subgroups in Common Variable Immunodeficiency. Blood.

[17] Abolhassani H., Amirkashani D., Parvaneh N., Mohammadinejad P., Gharib B., Shahinpour S., Hirbod-Mobarakeh A., Mirghorbani M., Movahedi M., Gharagozlou M. (2013). Autoimmune Phenotype in Patients with Common Variable Immunodeficiency. J. Investig. Allergol. Clin. Immunol..

[18] Edwards E.S.J., Bosco J.J., Aui P.M., Stirling R.G., Cameron P.U., Chatelier J., Hore-Lacy F., O’Hehir R.E., van Zelm M.C. (2019). Predominantly Antibody-Deficient Patients With Non-Infectious Complications Have Reduced Naive B, Treg, Th17, and Tfh17 Cells. Front. Immunol..

[19] Warnatz K., Schlesier M. (2008). Flowcytometric Phenotyping of Common Variable Immunodeficiency. Cytom. B Clin. Cytom..

[20] Giovannetti A., Pierdominici M., Mazzetta F., Marziali M., Renzi C., Mileo A.M., De Felice M., Mora B., Esposito A., Carello R. (2007). Unravelling the Complexity of T Cell Abnormalities in Common Variable Immunodeficiency. J. Immunol..

[21] Bateman E.A.L., Ayers L., Sadler R., Lucas M., Roberts C., Woods A., Packwood K., Burden J., Harrison D., Kaenzig N. (2012). T Cell Phenotypes in Patients with Common Variable Immunodeficiency Disorders: Associations with Clinical Phenotypes in Comparison with Other Groups with Recurrent Infections. Clin. Exp. Immunol..

[22] von Spee-Mayer C., Koemm V., Wehr C., Goldacker S., Kindle G., Bulashevska A., Proietti M., Grimbacher B., Ehl S., Warnatz K. (2019). Evaluating Laboratory Criteria for Combined Immunodeficiency in Adult Patients Diagnosed with Common Variable Immunodeficiency. Clin. Immunol..

[23] Azizi G., Rezaei N., Kiaee F., Tavakolinia N., Yazdani R., Mirshafiey A., Aghamohammadi A. (2016). T-Cell Abnormalities in Common Variable Immunodeficiency. J. Investig. Allergol. Clin. Immunol..

[24] Unger S., Seidl M., van Schouwenburg P., Rakhmanov M., Bulashevska A., Frede N., Grimbacher B., Pfeiffer J., Schrenk K., Munoz L. (2018). The TH1 Phenotype of Follicular Helper T Cells Indicates an IFN-γ-Associated Immune Dysregulation in Patients with CD21low Common Variable Immunodeficiency. J. Allergy Clin. Immunol..

[25] Le Saos-Patrinos C., Loizon S., Blanco P., Viallard J.-F., Duluc D. (2020). Functions of Tfh Cells in Common Variable Immunodeficiency. Front. Immunol..

[26] Turpin D., Furudoi A., Parrens M., Blanco P., Viallard J.-F., Duluc D. (2018). Increase of Follicular Helper T Cells Skewed toward a Th1 Profile in CVID Patients with Non-Infectious Clinical Complications. Clin. Immunol..

[27] Robinson G.A., Peng J., Dönnes P., Coelewij L., Naja M., Radziszewska A., Wincup C., Peckham H., Isenberg D.A., Ioannou Y. (2020). Disease-Associated and Patient-Specific Immune Cell Signatures in Juvenile-Onset Systemic Lupus Erythematosus: Patient Stratification Using a Machine-Learning Approach. Lancet Rheumatol..

[28] Jin W., Luo Z., Yang H. (2020). Peripheral B Cell Subsets in Autoimmune Diseases: Clinical Implications and Effects of B Cell-Targeted Therapies. J. Immunol. Res..

[29] Wehr C., Eibel H., Masilamani M., Illges H., Schlesier M., Peter H.-H., Warnatz K. (2004). A New CD21low B Cell Population in the Peripheral Blood of Patients with SLE. Clin. Immunol..

[30] Jablonka A., Etemadi H., Adriawan I.R., Ernst D., Jacobs R., Buyny S., Witte T., Schmidt R.E., Atschekzei F., Sogkas G. (2020). Peripheral Blood Lymphocyte Phenotype Differentiates Secondary Antibody Deficiency in Rheumatic Disease from Primary Antibody Deficiency. J. Clin. Med..

[31] Wirsum C., Glaser C., Gutenberger S., Keller B., Unger S., Voll R.E., Vach W., Ness T., Warnatz K. (2016). Secondary Antibody Deficiency in Glucocorticoid Therapy Clearly Differs from Primary Antibody Deficiency. J. Clin. Immunol..

[32] ESID Registry—Working Definitions for Clinical Diagnosis. https://Esid.Org/Education/Diagnostic-Criteria-Pid.

[33] Chapel H., Lucas M., Lee M., Bjorkander J., Webster D., Grimbacher B., Fieschi C., Thon V., Abedi M.R., Hammarstrom L. (2008). Common Variable Immunodeficiency Disorders: Division into Distinct Clinical Phenotypes. Blood.

[34] Petri M., Orbai A.-M., Alarcón G.S., Gordon C., Merrill J.T., Fortin P.R., Bruce I.N., Isenberg D., Wallace D.J., Nived O. (2012). Derivation and Validation of the Systemic Lupus International Collaborating Clinics Classification Criteria for Systemic Lupus Erythematosus. Arthritis Rheum..

[35] Boldt A., Borte S., Fricke S., Kentouche K., Emmrich F., Borte M., Kahlenberg F., Sack U. (2014). Eight-color immunophenotyping of T-, B-, and NK-cell subpopulations for characterization of chronic immunodeficiencies. Cytom. B Clin. Cytom..

[36] Fevang B., Yndestad A., Sandberg W.J., Holm A.M., Müller F., Aukrust P., Frøland S.S. (2007). Low Numbers of Regulatory T Cells in Common Variable Immunodeficiency: Association with Chronic Inflammation in Vivo. Clin. Exp. Immunol..

[37] Kofod-Olsen E., Jørgensen S.E., Nissen S.K., Westh L., Møller B.K., Østergaard L., Larsen C.S., Mogensen T.H. (2016). Altered Fraction of Regulatory B and T Cells Is Correlated with Autoimmune Phenomena and Splenomegaly in Patients with CVID. Clin. Immunol..

[38] Arandi N., Mirshafiey A., Jeddi-Tehrani M., Abolhassani H., Sadeghi B., Mirminachi B., Shaghaghi M., Aghamohammadi A. (2013). Evaluation of CD4+CD25+FOXP3+ Regulatory T Cells Function in Patients with Common Variable Immunodeficiency. Cell. Immunol..

[39] Kutukculer N., Azarsiz E., Aksu G., Karaca N.E. (2016). CD4+CD25+Foxp3+ T Regulatory Cells, Th1 (CCR5, IL-2, IFN-γ) and Th2 (CCR4, IL-4, Il-13) Type Chemokine Receptors and Intracellular Cytokines in Children with Common Variable Immunodeficiency. Int. J. Immunopathol. Pharmacol..

[40] Ebbo M., Gérard L., Carpentier S., Vély F., Cypowyj S., Farnarier C., Vince N., Malphettes M., Fieschi C., Oksenhendler E. (2016). Low Circulating Natural Killer Cell Counts Are Associated With Severe Disease in Patients With Common Variable Immunodeficiency. EBioMedicine.

[41] Wehr C. (2016). Trying to Understand NK Cell Function in Vivo Points towards a Severity Score for CVID Patients. EBioMedicine.

[42] Haymore B.R., Mikita C.P., Tsokos G.C. (2008). Common Variable Immune Deficiency (CVID) Presenting as an Autoimmune Disease: Role of Memory B Cells. Autoimmun. Rev..

[43] Barbosa R.R., Silva S.P., Silva S.L., Melo A.C., Pedro E., Barbosa M.P., Pereira-Santos M.C., Victorino R.M.M., Sousa A.E. (2011). Primary B-Cell Deficiencies Reveal a Link between Human IL-17-Producing CD4 T-Cell Homeostasis and B-Cell Differentiation. PLoS ONE.

[44] Catalán D., Mansilla M.A., Ferrier A., Soto L., Oleinika K., Aguillón J.C., Aravena O. (2021). Immunosuppressive Mechanisms of Regulatory B Cells. Front. Immunol..

[45] Bosma A., Abdel-Gadir A., Isenberg D.A., Jury E.C., Mauri C. (2012). Lipid-antigen presentation by CD1d(+) B cells is essential for the maintenance of invariant natural killer T cells. Immunity.

[46] Chekol Abebe E., Asmamaw Dejenie T., Mengie Ayele T., Dagnew Baye N., Agegnehu Teshome A., Tilahun Muche Z. (2021). The Role of Regulatory B Cells in Health and Diseases: A Systemic Review. J. Inflamm. Res..

[47] Yesillik S., Agrawal S., Gollapudi S.V., Gupta S. (2019). Phenotypic Analysis of CD4+ Treg, CD8+ Treg, and Breg Cells in Adult Common Variable Immunodeficiency Patients. Int. Arch. Allergy Immunol..

[48] Barsotti N.S., Almeida R.R., Costa P.R., Barros M.T., Kalil J., Kokron C.M. (2016). IL-10-Producing Regulatory B Cells Are Decreased in Patients with Common Variable Immunodeficiency. PLoS ONE.

[49] Stuchlý J., Kanderová V., Vlková M., Heřmanová I., Slámová L., Pelák O., Taraldsrud E., Jílek D., Králíc Ková P., Fevang B. (2017). Common Variable Immunodeficiency Patients with a Phenotypic Profile of Immunosenescence Present with Thrombocytopenia. Sci. Rep..

[50] Koga T., Ichinose K., Kawakami A., Tsokos G.C. (2020). Current Insights and Future Prospects for Targeting IL-17 to Treat Patients With Systemic Lupus Erythematosus. Front. Immunol..

[51] Osnes L.T., Nakken B., Bodolay E., Szodoray P. (2013). Assessment of Intracellular Cytokines and Regulatory Cells in Patients with Autoimmune Diseases and Primary Immunodeficiencies—Novel Tool for Diagnostics and Patient Follow-Up. Autoimmun. Rev..

