# Supplementary material for: Patients with Common Variable Immunodeficiency Complicated by Autoimmune Phenomena Have Lymphopenia and Reduced Treg, Th17, and NK Cells
Source: J Clin Med. 2021 Jul 29;10(15):3356. doi: 10.3390/jcm10153356 (PMC8348468; doi:10.3390/jcm10153356)
Supplement: Supplementary file 1 [file jcm-10-03356-s001.zip › jcm-1255757-supplementary.pdf]

Table 1S. Internal laboratory reference values of lymphocyte subpopulations and absolute numbers

	% of lymphocytes	cells/µl
lymphocytes T (CD3+)	60 – 82	900 – 2100
lymphocytes Th (CD3+ CD4+)	33 – 55	500 – 1300
lymphocytes Ts (CD3+ CD8+)	18 – 40	280 – 900
CD4/CD8 ratio	1.1 – 2.8	
NK cells (CD3- CD16+ CD56+)	7 – 23	90 – 630
lymphocytes B (CD19+)	5 – 16	120 – 400

Table 2S. Internal laboratory reference values if the proportions of B lymphocyte maturation

	% CD19+
Transitional B cells<br>IgM++ IgD++ CD38++ CD27- CD21+	0.9 – 6.3
Naive B cells<br>IgM+ IgD++ CD38+ CD27- CD21+	53.3 – 86.0
Non-switched memory B cells (Marginal zone-like B cells)<br>IgM++ IgD+ CD38+ CD27+ CD21+	3.3 – 12.8
Class switched memory B cells<br>IgM- IgD- CD38+ CD27+ CD21+	4.0 – 22.1
$CD21^{low}$ B cells<br>IgM+ IgD+ $CD38^{low}$ CD27- $CD21^{low}$	0.6 – 7.6
Plasmablasts<br>IgM-/+ IgD- CD38+++ CD27++ CD21+	0.1 – 1.5

Table 3S. Internal laboratory reference values if the proportions of T lymphocyte maturation

	% CD4+	% CD8+
Naive T cells<br>CD45RA+ CD197+	18.2 – 53.2	18.6 – 70.6
Effector T cells<br>CD45RA+ CD197-	1.1 – 9.0	4.0 – 24.0
Central memory T cells<br>CD45RO+ CD197+	8.3 – 27.1	0,5 – 5,4%

Effector memory T cells<br>CD45RO+ CD197-	17.2 – 51.4	16.7 – 42.6	
Th17<br>CD45RO+ CD196+	10.8 – 39.0	-	-
Recent thymic emigrants T cells<br>CD45RA+ CD62L+ CD31+	14.4 – 38.3	16.7 – 64.7	

Figure 1S. Representative gating strategy of main lymphocytes subpopulation and T regulatory (Tregs), B regulatory cells (B regs) and Th17 cells in study group.

(A) T (CD4+ and CD8+ cells), B, NK lymphocytes gating strategy: FSC-A vs. FSC-H plot: Gating the cells that have an equal area and height, thus removing clumps (greater FSC-A relative to FSC-H) and debris (very low FSC), CD45 vs. SSC-A plot: Selection of lymphocytes based on their SSC/CD45+ properties, CD3 vs. CD19 plot: Selection of lymphocytes T (pink and yellow) and B (orange) based on their CD3+ or CD19+ properties, CD4 vs. CD8 plot: Selection of subpopulation of lymphocytes T: CD4 cells (pink) and CD8 cells (yellow) based on their CD4+ or CD8+ properties, CD3 vs. CD16 plot: Selection of NK cell (blue) based on their CD3-/CD16+ properties

(B) Representative dot plots of Tregs, Bregs and Th17 cells (green), CD25 vs. 127 plot: Selection of Tregs (white) based on their $CD25^{high}$/CD127- properties, CD5 vs. CD1d plot: Selection of Breg (light blue) based on their CD5+/CD1d+ properties, CD196 vs. CD45RO plot: Selection of Th17 cells (green) based on their CD196+/CD45RO+ properties.

A

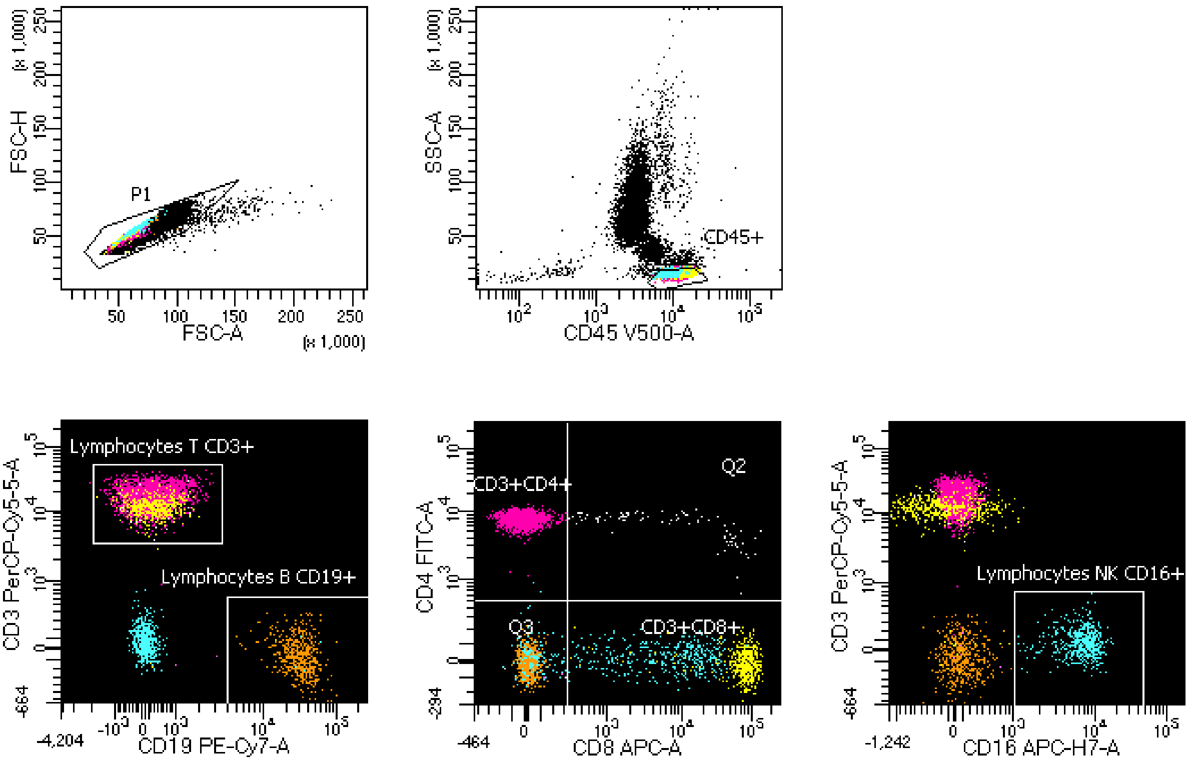
FSC-H
(x 1,000)
P1
FSC-A
(x 1,000)
SSC-A
(x 1,000)
CD45+
CD45 V500-A
Lymphocytes T CD3+
Lymphocytes B CD19+
CD3 PerCP-Cy5-5-A
CD19 PE-Cy7-A
CD3+CD4+
Q2
Q3
CD3+CD8+
CD4 FITC-A
CD8 APC-A
Lymphocytes NK CD16+
CD3 PerCP-Cy5-5-A
CD16 APC-H7-A

B

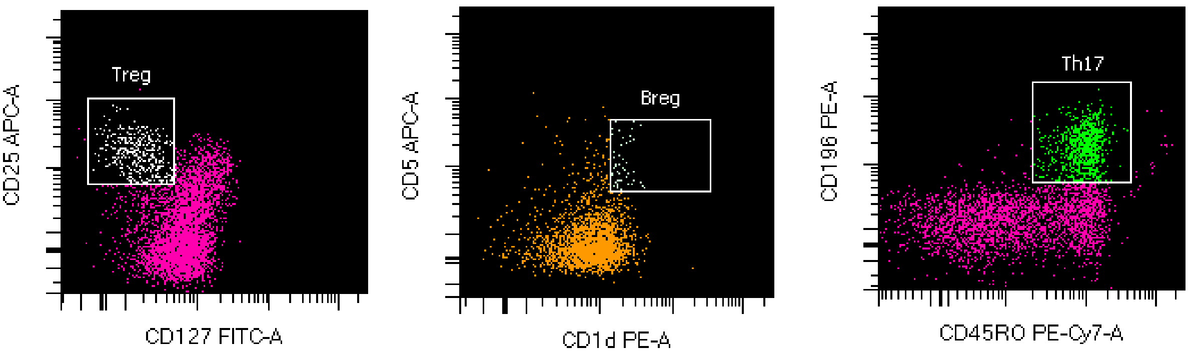
Treg
CD25 APC-A
CD127 FITC-A
Breg
CD5 APC-A
CD1d PE-A
Th17
CD196 PE-A
CD45RO PE-Cy7-A

Figure 2S. Representative B lymphocytes maturation gating strategy in study group. (A) B lymphocytes gating strategy: FSC-A vs. FSC-H plot: Gating the cells that have an equal area and height, thus removing clumps (greater FSC-A relative to FSC-H) and debris (very low FSC), CD45 vs. SSC-A plot: Selection of lymphocytes based on their SSC/CD45+ properties, CD19 vs. SSC-A plot: Selection of lymphocytes B based on their SSC/CD19+ properties. (B) Representative dot plots of each maturation B subsets cells: transitional B cells (orange), naïve B cells (blue), non-switched memory B cells (red), class switched memory B cells (yellow), CD21^{low} cells (purple) and plasmablasts (green) based on their IgD/IgM, IgD/CD38, CD21/CD38, IgM/CD38, CD27/CD21 properties (phenotypes of all cells described in section: material and method).

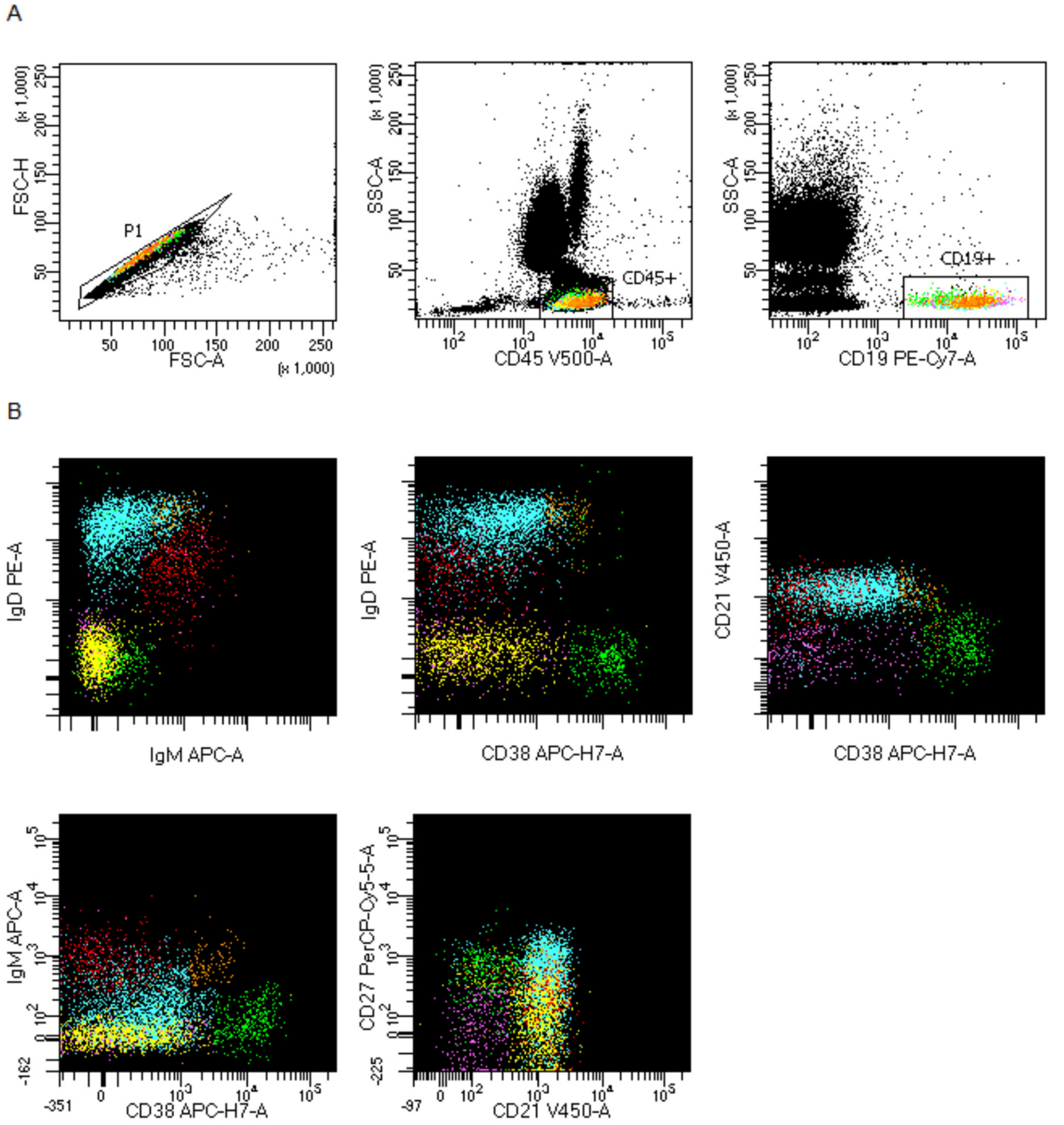

Figure 3S. Representative T lymphocytes maturation gating strategy in study group. (A) T lymphocytes gating strategy: FSC-A vs. FSC-H plot: Gating the cells that have an equal area and height, thus removing clumps (greater FSC-A relative to FSC-H) and debris (very low FSC), CD45 vs. SSC-A plot: Selection of lymphocytes based on their SSC/CD45+ properties, CD3 vs. SSC-A plot: Selection of lymphocytes T based on their SSC/CD3+ properties. CD4 vs. CD8 plot: Selection of lymphocytes T CD4+ (pink) and CD8+ (yellow) based on their CD4+ or CD8+ properties. (B) Representative dot plots of each maturation T CD4+ subsets cells: recent thymic emigrants T CD4+ cells, naïve CD4+ T cells, effector CD4+ T cells, central memory CD4+ T cells and effector memory CD4+ T cells based on CD197/CD45RO, CD62L/CD45RA, CD45RA/CD31 properties (phenotypes of all cells described in section: material and method). **(C)** Representative dot plots of each maturation T CD8+ subsets cells: recent thymic emigrants T CD8+ cells, naïve CD8+ T cells, effector CD8+ T cells, central memory CD8+ T cells and effector memory CD8+ T cells based on their CD197/CD45RO, CD62L/CD45RA, CD45RA/CD31 properties (phenotypes of all cells described in section: material and method).

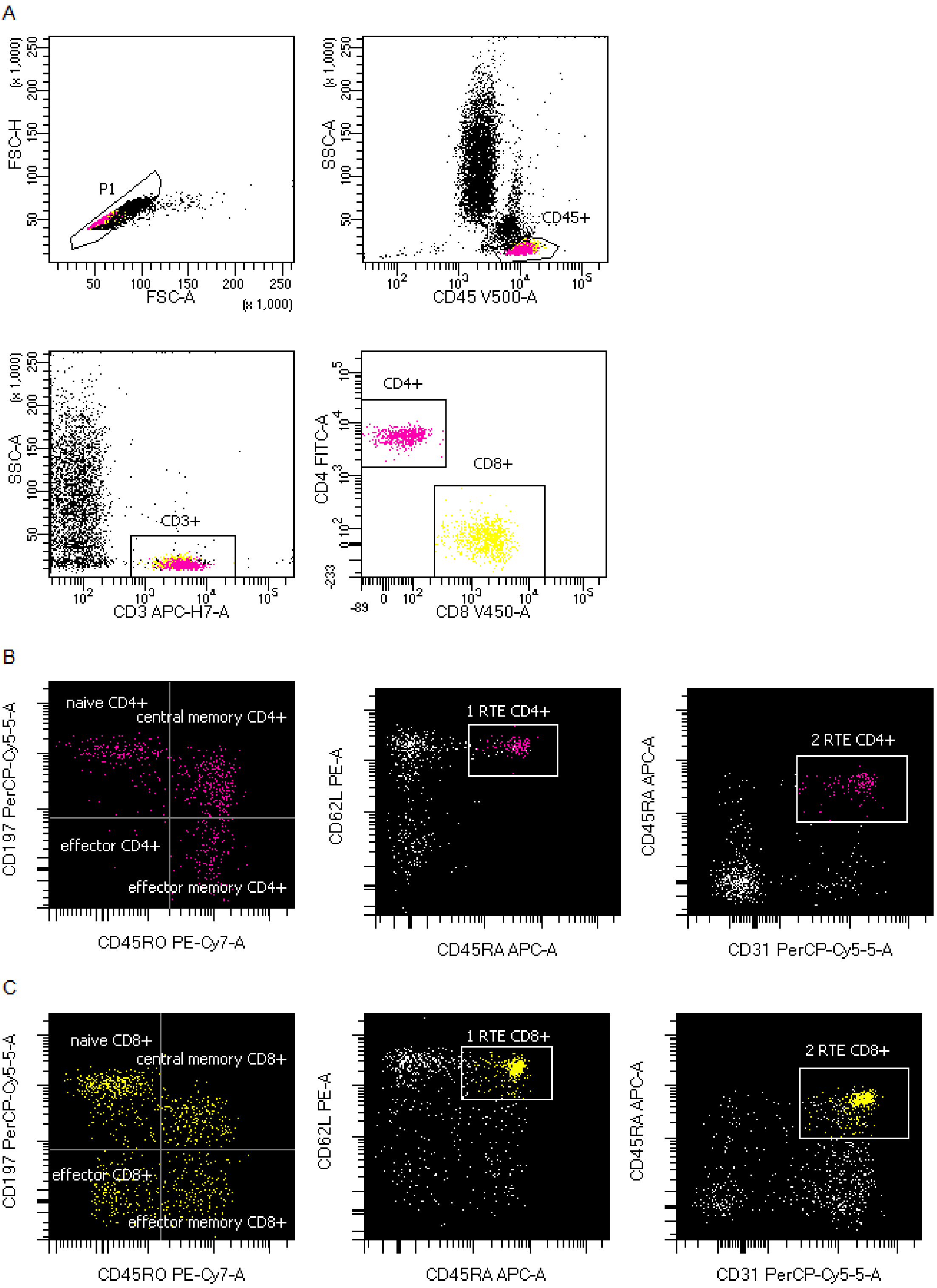
A
P1
FSC-H (x 1,000)
FSC-A (x 1,000)
CD45+
SSC-A (x 1,000)
CD45 V500-A
CD3+
SSC-A (x 1,000)
CD3 APC-H7-A
CD4+
CD8+
CD4 FITC-A
CD8 V450-A
B
naive CD4+
central memory CD4+
effector CD4+
effector memory CD4+
CD197 PerCP-Cy5-5-A
CD45RO PE-Cy7-A
1 RTE CD4+
CD62L PE-A
CD45RA APC-A
2 RTE CD4+
CD45RA APC-A
CD31 PerCP-Cy5-5-A
C
naive CD8+
central memory CD8+
effector CD8+
effector memory CD8+
CD197 PerCP-Cy5-5-A
CD45RO PE-Cy7-A
1 RTE CD8+
CD62L PE-A
CD45RA APC-A
2 RTE CD8+
CD45RA APC-A
CD31 PerCP-Cy5-5-A
